# Evaluation of the Relationship between Adipose Metabolism Patterns and Secretion of Appetite-Related Endocrines on Chicken

**DOI:** 10.3390/ani10081282

**Published:** 2020-07-27

**Authors:** Wen Yang Chuang, Yun Chen Hsieh, Li Wei Chen, Tzu-Tai Lee

**Affiliations:** 1 Department of Animal Science, National Chung Hsing University, Taichung 402, Taiwan; xssaazxssaaz@yahoo.com.tw (W.Y.C.); richard840909@gmail.com (Y.C.H.); dachch4@gmail.com (L.W.C.); 2 The iEGG and Animal Biotechnology Center, National Chung Hsing University, Taichung 402, Taiwan

**Keywords:** adipose metabolism, appetite, feed composition, poultry endocrines

## Abstract

**Simple Summary:**

The weight of an animal conforms to a certain growth pattern. Among others, feed, environment, and body composition, in addition to genetics, affect the animal’s feed consumption and body weight. Under normal circumstances, the body weight of an animal is mainly affected by feed intake, and body composition may significantly influence feed intake. Therefore, this report sets out the effects of fat accumulation on lipid metabolism and appetite, and finally introduces the effects of feeding patterns on animal feed intake.

**Abstract:**

In addition to the influence of genes, the quality of poultry products is mainly controlled by the rearing environment or feed composition during rearing, and has to meet human use and economical needs. As the only source of energy for poultry, feed considerably affects the metabolic pattern of poultry and further affects the regulation of appetite-related endocrine secretion in poultry. Under normal circumstances, the accumulation of lipid in adipose reduces feed intake in poultry and increases the rate of adipose metabolism. When the adipose content in cells decreases, endocrines that promote food intake are secreted and increase nutrient concentrations in serum and cells. By regulating the balance between appetite and adipose metabolism, the poultry’s growth and posture can maintain a balanced state. In addition, increasing fiber composition in feed can effectively increase poultry welfare, body weight, lean composition and antioxidant levels in poultry. According to this, the concept that proper fiber content should be added to feed should be considered for better economic benefits, poultry welfare and meat productivity.

## 1. Introduction

Feeding is the most important way for animals to obtain energy, and use this energy to sustain life. Energy intake through the diet mainly provides the animals with growth and maintenance of basal metabolism, but excessive energy intake can lead to obesity. In general, animals have a complex endocrine system that regulates the total amount of food consumed to avoid obesity [1]. However, environmental stresses such as heat stress, microbial infections, or inappropriate day and night rhythms may cause changes in animal feeding patterns [2,3]. Excessive adipose accumulation can cause prolonged chronic inflammation in animals which may lead to cardiovascular disease and elevated cortisol, increasing mortality in animals [4,5]. In addition, excessive weight may also cause lesions in the feet and reduce the animal’s immunity [6,7]. Moreover, modern consumers are pursuing a healthy low-fat diet, meat which accumulates excessive fat is not popular. Poultry contributes at least one third of the output value in terms of economical animals. There are many previous studies that have only explored the relationship between appetite regulation and obesity in mammals, such as pigs and mice, but the organs and physiological structures of poultry are not the same as those of mammals, and the degree of response to endocrine secretions also varies. Therefore, poultry and mammals must be discussed separately [8].

As corn and soybean meal are the major components of poultry diets, the feed is closely associated to the accumulation of abdominal fat and also alters the microbiota and further changes the intestinal epithelial cell health [9,10,11]. The microbiota composition also affects the nutrition absorption and utilization and further changes the concentration of serum nutrition level. High nutrition levels increase adipose storage in animal cells thereby changing the poultry appetite [9]. By the circulation mentioned above, feed intake patterns can strengthen or weaken the health of animals. In addition, many plant extracts, dietary fiber, probiotics, and prebiotics can improve the efficiency of nutrient utilization by animals, change carcass properties or improve antioxidant and immune levels by changing the intestinal environment or enzyme system [12,13,14,15].

In order to consider animal welfare, antibiotic-free rearing, reasonable costs and consumer preferences, the development of the poultry industry enhanced by feed composition to regulate the appetite and adipose metabolism patterns is farsighted. Moreover, the obesity of poultry is correlated with mortality and dressing percentage [16,17]. Therefore, this review article aims to discuss how factors such as endocrine secretions, fat accumulation, and dietary patterns affect the appetite of poultry, and further discuss its impact on the welfare of poultry.

## 2. Factors on Appetite Regulation

**Figure 1:**
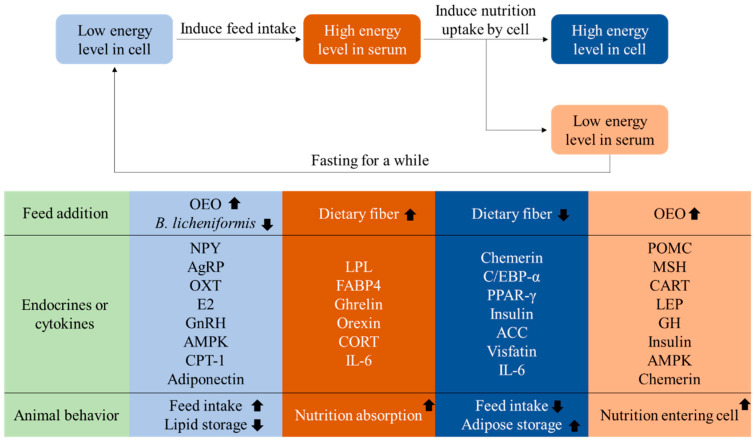
The potential impact factors controlling cell and serum energy states. ACC: acetyl-CoA carboxylase; AgRP: agouti-related protein; AMPK: AMP-activated protein kinase; C/EBPα: CCAAT/enhancer-binding protein alpha; CART: cocaine and amphetamine regulated transcript; CORT: corticosterone; CPT-1: carnitine palmitoyltransferase-1; E2: Estrodiol; FABP4: fatty-acid-binding proteins 4; GH: growth hormone; GnRH: gonadotropin-releasing hormone; IL-6: Interleukin-6; LEP: leptin; LPL: lipoprotein lipase; MSH: melanocyte stimulating hormone; OEO: oregano essential oil; OXT: oxytocin; POMC: pro-opiomelanocortin; PPAR-γ: peroxisome proliferator activated receptor γ.

Song et al. [18] reported the differences in energy balance and endocrines in mammals and poultry, they also pointed out that animal appetite regulation was mainly through the central nervous system. Recent researchers further indicated that endocrines such as leptin (LEP), reproductive-related hormone, insulin, and AMP-activated protein kinase (AMPK), which were modulated in accordance with the energy level in the diet, were also positively related to the adipose metabolism [19]. Furthermore, by the recent research, we could find that although appetite-related endocrines could significantly influence the animal appetite, the energy balance-related endocrines still played a very important role in adjusting animal appetite by the regulation of both adipolysis and adipogenesis-related protein expression [1,20]. In normal conditions, animals store extra energy in the adipocyte and decrease feed intake when energy levels in cells are high [1]. However, after a few hours fasting, the source for producing energy is degraded, and therefore decreases the energy levels in cells, thereby starting to increase feed intake again [1]. After eating, animal serum nutrient concentration increases from the nutrients absorbed from feed, and high nutrient concentration induces the nutrient uptake by animal cells [9]. Within this eat or not eat circulation, many different kinds of endocrines are involved and alter body composition, energy level, feed intake amount, etc., in the absence of any endocrine secretions. As far as is known, there are several complex endocrines involved in the regulation of poultry appetite, including but not limited to cocaine and amphetamine regulated transcript (CART), pro-opiomelanocortin (POMC), agouti-related protein (AgRP), neuropeptide Y receptor (NPY), AMPK, orexin and ghrelin which play a role in the regulation of appetite in acute heat-exposed broiler chickens and their upstream and downstream genes [9,20,21,22]. Genes related to fat metabolism in poultry include, but are not limited to, carnitine palmitoyltransferase (CPT-1), chemerin, visfatin, CCAAT/enhancer-binding protein alpha (C/EBP-α), AMPK, adipose triglyceride lipase (ATGL), and interleukin 6 (IL-6) etc. [4,23]. According to previous researchers, the possible mechanism of endocrine regulation of poultry appetite is shown in Figure 1. The effects of these factors on animal appetite regulation and energy metabolism are fully described in the following sections.

## 3. The Endocrine Regulation of the Avian Appetite

### 3.1. The Pro-Opiomelanocortin (POMC)/Cocaine and Amphetamine Regulated Transcript (CART) and Neuropeptide Y (NPY)/Agouti-Related Protein (AgRP) Regulation of the Avian Appetite

Animals have developed endocrines that adjust their physiological functions in accordance with the day and night rhythm [3]. One of the most common endocrines is POMC. POMC/CART and NPY/AgRP are the protein fragments produced by the arcuate nucleus in the hypothalamus [24,25]. POMC is secreted by the pituitary gland and can be cut into many fragments, including the melanocyte stimulating hormone (MSH) family (relating to appetite), adrenocorticotropic hormone (ACTH, relating to energy steady stage), β-Endorphin and encephalin (relating to stress regulation), etc., [24]. The mutation of POMC may cause obesity and renal insufficiency [24]. Broiler chickens have a naturally higher feed intake than laying hens and have a much higher meat production. There is no significant difference in POMC mRNA content in the hypothalamus of broilers and layers, but the application of β-MSH can only reduce the appetite of laying hens. Therefore, the lack of β-MSH-mediated anorexia in broilers may be related to the increase in food intake [21]. MSH can significantly suppress appetite in mammals, but its role in poultry is unclear. Honda et al. [21] pointed out that α-MSH can significantly reduce the feed intake of laying hens and broilers. Intracerebroventricular injection of α-MSH would decrease the neuropeptide Y receptor 1 (NPYR1) mRNA and increase c-Fos mRNA expression in the paraventricular nucleus (PVN), indicating that there is some interaction between the NPY and α-MSH [25]. At the same time, the appetite suppressed by α-MSH is restored within 1 h with the increase of NPY, oxytocin receptor (OXTR), and AgRP mRNA expression levels [25]. Similar to POMC, CART can be split into two smaller peptides, which inhibit animal appetite and stimulate animal activity [24].

In contrast to POMC/CART, NPY/AgRP can promote appetite and is negatively correlated with leptin performance [24]. NPY can increase the mRNA expression of fatty acid binding protein 4 (FABP4) and lipoprotein lipase (LPL), and decrease C/EBPα and peroxisome proliferator activated receptor γ (PPARγ) in chicken adipocyte [23]. The same results are shown in in vivo tests [26]. In addition, injection of 2 nmol NPY can increase the Hubbard X Cobb 500 broiler’s intake of a high-carbohydrate and high-protein diet, and high-carbohydrate diets can also increase NPY-stimulated high-fat diet intake [9]. The NPY and NPYR in 1 to 8-day-old chunky broilers are significantly lower than in Leghorn layer chicks; however, the intracerebroventricular NPY (0.2–0.4 μg) would increase the feed intake in both broiler and layer chicks [27]. There is also evidence that NPY has a better appetite-promotion effect in higher weight chickens [26,28]. According to the above results, it is known that NPY can increase the feed intake of poultry and reduce the adipolysis [26,27]. AgRP is cosecreted with NPY, which can promote appetite and reduce metabolism. LEP can inhibit the release of AgRP, while ghrelin can promote the release of NPY/AgRP. AgRP promotes ACTH and cortisol release and inhibits thyrotropin-releasing hormone (TRH) activity [29].

### 3.2. The Leptin Regulation of the Avian Appetite

LEP is one of the major appetite regulation factors in mammals and causes mouse obesity when LEP is absent. Although the leptin receptor (LEPR) had been noted in Horev’s et al. [30] report, the existence of LEP in avians was discussed for many decades until Seroussi et al. [31] discovered the existence of avian LEP. Murase et al. [32] indicated that LEP could induce growth hormone (GH) mRNA expression, and thereby improve muscle growth and lipid metabolism. In avians, leptin can promote the chicken growth hormone (cGH) gene expression in chicken-LEPR-expressed chinese hamster ovary cells when the chPit-1α is present. The expression mentioned above was not a signal transducer and activator of transcription (STAT)-dependent but janus kinase 2 (JAK2)-dependent (p42/44 MAPK and PI3K pathway, included). An in ovo injection of 0.5 or 5 μg LEP into Sanhuang broiler breeder eggs could increase the average daily gain and decrease the expression of hypothalamic glucocorticoid receptor (GR) of chicks [33]. The LEPR of broilers decreases and the GH receptor when the broilers’ age increases [34]. Denbow et al. [35] indicated that leptin (2.5–10 μg) dose-dependently decreases the feed intake of broilers (4-week-old) and Leghorns (7-week-old) by the intracerebroventricular (i.c.v.) injection. Among them, Leghorn layers are more sensitive to LEP and reduce much more feed intake with the LEP injection, than broilers [36].

Lei et al. [37] indicated that injection of LEPR antibodies (7.5 or 15 mg polyclonal anti-LEPR and 15 mg monoclonal anti-LEPR) can increase the feed intake of Chinese indigenous Gushi pullets, and the effect of monoclonal anti-LEPR is better. After injection with anti-LEPR, the levels of glucose, triglycerides (TG), high density lipoprotein (HDL), low density lipoprotein (LDL) and cholesterol in Gushi pullets’ serum decreased. Injection of antibodies also causes a slight decrease in POMC and melanocortin 4 receptor, and LEPR in the hypothalamus (no significant difference), but it upregulates the performance of LEPR in the liver, abdominal fat and breast muscles, and increases the STAT3 phosphate in the liver. In summary, anti-LEPR indirectly enhances muscle formation and fat metabolism by inhibiting LEPR performance (at 30 days old). However, Sims et al. [38] indicated that intracerebroventricular injection of chicken leptin (0.3–3 nmol) in the fasted 180 min Cobb broilers (4 days old) did not significantly affect the food and water intake and the behaviors of broilers. 

Seroussi et al. [31] indicated that chicken LEP was highly correlated with the expression of LEPR (r2 = 0.86), while chicken LEP could be detected in the hypothalamus, cerebellum, pituitary, pancreas and testis, but hardly in the liver and adipocyte. In addition, because the LEP was undetected in chicken serum, Seroussi et al. [31] also assume that LEP may only affect the physiological response of chickens through autocrine/paracrine, which is also different from mammals to convey LEP by circulating. The LEPR of chickens is mostly long form, and this seems to be different from the short form common in mammals [31]. Accordingly, there are several differences in the function of LEP between chickens and mammals.

In conclusion, according to research mentioned above [31,32,33,34,35,36,37,38], the current evidence shows that there is indeed a functional leptin in avians. Leptin inhibits poultry appetite mainly by inhibiting NPY and AgRP and upregulating the expression of POMC and α-MSH. However, LEP can also phosphorylate STAT3, p42/44 MAPK and PI3K pathway and stimulate cGH production. cGH further enhances the adipose metabolism and muscle formation and reduces the concentrations of glucose and TG in the serum, and promotes the production of NPY and AgRP in the inferior colliculus and increases the feed intake of chickens (Figure 1). Although there is evidence that leptin has no effect on the feed intake of poultry, it may be related to the dose used or the age of the birds. However, further research is necessary on LEP injection methods, dosage, and chicken age to confirm the role of LEP in chickens.

### 3.3. The Reproductive Hormone Regulation of the Avian Appetite

In mammals, estrogen can regulate the animal’s physiological cycle and affect energy metabolism. However, due to different reproductive patterns, the function of estrogen may be different between poultry and mammals. This section discusses the known effects of estrogen on energy metabolism in poultry.

#### 3.3.1. Oxytocin

Like mammalian oxytocin (OXT), mesotocin is one kind of OXT for poultry, and it is only one amino acid away from the OXT of mammals. In mammals, OXT can reduce animal vitality and feed intake and increase adipose metabolism in adipocyte and myotube differential [39]. Intracerebroventricular injection of oxytocin could significantly decrease the water and feed intake for at least 180 min and increase the c-Fos immunoreaction in most of the parts of hypothalamus [40]. The results mentioned above were corticotropin-releasing factor (CRF) dependent and could also increase the adipolysis-related mRNA expression [40,41].

#### 3.3.2. Estradiol

Estradiol (E2) is one of the steroid endocrines secreted by the ovary and regulates the maturity of follicles and metabolism in mammals. There is a similar effect of the E2 on the avian; however, to the best of our knowledge, there are only a few papers which have discussed the relationship between E2 and energy metabolism. Ren et al. [42] indicated that E2 does not affect the expression of melanocortin receptor 5 (MC5R, one of the receptors of MSH, which can increase adipolysis) but decreases the expression of PPARγ and thereby increases the melanocortin receptor accessory protein (MRAP) expression. Therefore, we can speculate that E2 can increase the energy metabolism and thereby maintain energy balance [42,43].

#### 3.3.3. Gonadotropin-Releasing Hormone (GnRH), Luteinizing Hormone (LH) and Follicle Stimulating Hormone (FSH)

Gonadotropin-releasing hormone (GnRH) is a very common reproductive-related hormone, which can induce the expression of follicle-stimulating hormone (FSH) and luteinizing hormone (LH). To the best of our knowledge, there is little published research about the relationship between GnRH, LH or FSH and energy metabolism. However, there is a study of gonadotropin inhibitory hormone (GnIH). In contrast to GnRH, GnIH inhibits FSH and LH secretion. McConn et al. [39] indicate that GnIH would increase the feed intake by the increase of NPY and decrease of POMC. From the information mentioned above, we can speculate that GnRH, LH and FSH also induce adipolysis and increase the energy gain of the ovaries. 

### 3.4. Insulin

It is well-known that poultry have higher blood glucose levels and insulin resistance compared to mammals [44,45]. The high insulin tolerance occurs in avians because of the absence of glucose transporter 4 (GLUT4) compared to mammals. In early research, Langslow and Hales [46] indicated that adipolysis in chicken adipocyte was mainly adjusted by glucagon but not insulin. Although the function of insulin in avians is much weaker than in mammals, insulin seems to slightly regulate the glucose intake of chicken adipocyte by suppressing the expression of glucagon [47]. In the latest study, insulin can increase adipocyte glucose intake mainly by GLUT1 through the Akt dependent pathway [48]. Furthermore, high weight chicken is more sensitive to insulin than low weight chicken by the activation of GLUT1 and GLUT2 [49]. However, the insulin injection can decrease mRNA expression of NPY, NPYR1 and NPYR5 in low weight chicken less than in high weight chicken [50]. In addition, insulin injections also increase the CART, α-MSH and CRF mRNA levels and decrease the appetite of chicks [51]. Overall, according to current research, it is known that chicken insulin can increase cell glucose intake mainly by the GLUT1 and regulate chicken appetite, especially in the low weight chicken. 

### 3.5. Ghrelin

Ghrelin is a common peptide in the vertebrate, which is synthesized by intestinal mucus and can induce growth hormone release and regulate animal appetite, lipid and glucose metabolism and reproductive function [52,53]. Although the function of ghrelin is similar in avians and mammals, the effect of ghrelin is species-specific. Ghrelin can induce the contraction of the chicken crop, proventriculus, ileum and colon, and speed up gastric emptying, but this function is negative with the age of chicken [52,53]. El-Magd et al. [54] indicated that the mRNA expression of the ghrelin receptor is positively related to chicken feed intake and can induce chicken growth hormone production.

Dexamethasone, a type of artificial corticosteroid medication, can decrease the expression of ghrelin in the duodenum, liver and abdominal fat but increase it in proventriculus and increase the ghrelin receptor in the duodenum, proventriculus and abdominal fat [8]. Song et al. [8] also indicated that although both insulin and glucocorticoid can increase the release of ghrelin, however, the glucose concentration in chicken serum seems not to affect chicken ghrelin expression level, which is in contrast with mammals.

### 3.6. Orexin

Orexin is expressed in the hypothalamus and participates in the regulation of animal energy homeostasis, which can increase feed intake in chickens [55]. Orexin can be secreted by avian muscle cells, and can be differentiated into two types, orexin A and orexin B [56]. Furthermore, orexin A can increase mitochondrial fission, but orexin B can increase mitochondrial fusion [56] and heat stress decreases the expression of orexin [2]. Although orexin has been found in poultry for decades, its function is still inconclusive. Current evidence shows that orexin plays an important role in chicken for meat production, and would regulate the ATP level in mitochondria thereby increasing or decreasing feed intake [55,56,57].

### 3.7. Hypothalamic-Pituitary-Adrenal Axis (HPA Axis)

Animals can resist stress through the HPA axis [5]. The hypothalamus can produce CRH to induce the pituitary to produce ACTH, and ACTH further promotes adrenal production of glucocorticoid (mainly corticosterone, which can increase feed intake) and thereby helping animals to withstand stress [5,58]. However, adrenaline or glucocorticoids help animals to cope with stress by regulating the animal’s serum glucose and fat metabolism patterns. When the energy in the animal is redistributed, the appetite and productivity of the animal is regulated [5]. Therefore, in recent years, research on the regulation of animal appetite by the HPA axis has been increasing.

As the final product of the HPA axis, corticosterone (CORT) can be used as a research indicator of energy homeostasis and appetite regulation. Studies have shown that CORT can increase chicken serum glucose levels and enhance lipolysis, but reduce muscle growth [59,60,61]. Although CORT can rapidly increase serum glucose and lipid levels to cope with stress, CORT can inhibit the growth of animals and cause fat accumulation in the pectoralis major muscle and abdominal fat of broilers by increasing cholesterol synthesis and uptake [59,61]. Furthermore, not only the muscle but also the growth of broilers is suppressed by CORT [62,63]. CORT downregulates the expression of nitric oxide synthase and suppresses insulin function to decrease glucose intake and differentiation in the muscle cells [64]. Subcutaneous injection (s.c.) injection of CORT increases serum glucose, insulin and cholesterol content and decreases the nonesterified fatty acids (NEFA), TG content and egg production in the Hy-line brown layer [65]. Furthermore, the content of ghrelin and CART increase and POMC and AgRP decrease in the hypothalamus of laying hens after injection of CORT [65]. However, under a stimulation of bacterial lipopolysaccharide, the content of TG decreases in Hy-line brown layer serum, but CORT can fix it [60]. In addition to these, CORT addition also increases the feed intake of low crude protein and carbohydrate diet, but decreases the feed intake of low lipid diet [66]. Overall, CORT can alter the level of serum glucose and lipid, and thereby enhance chicken selectivity of feed and decrease the feed intake and chicken growth. The function of appetite-related endocrines in poultry is shown in Table 1.

## 4. Poultry Adipose Accumulation and Its Impact on Poultry Health

### 4.1. Adipose Synthesis and Catabolism

The liver and adipose tissue are the two main sites of de novo fatty acid synthesis in higher vertebrates [69]. However, Leveille et al. [70] estimated the relative importance of adipose tissue and the liver as sites of fatty acid synthesis, chick adipose tissue was found to be of minor importance as compared to liver, accounting for no more than 30% of total fatty acid synthesis. Thus, the major site of fatty acid synthesis in chickens is the liver rather than the adipose tissue in mammals [70]. The liver catabolizes glucose to acetyl-CoA, and then produces fatty acids and cholesterol, which facilitate the packaging and release of very low-density lipoprotein (VLDL) into circulation [69,71].

**Figure 2:**
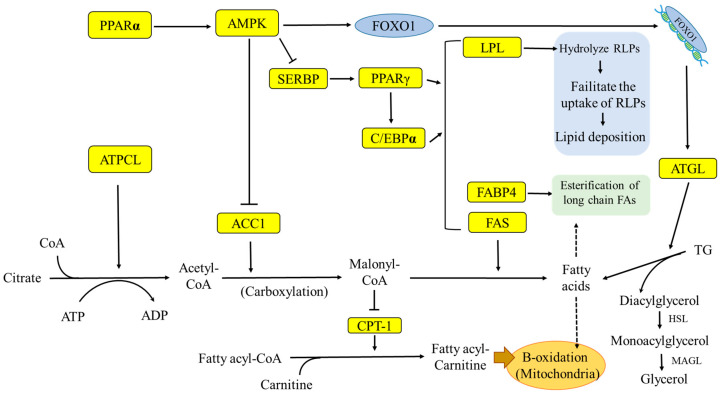
The key pathway of adipose metabolism. PPARα: peroxisome proliferator-activated receptor α; PPARγ: peroxisome proliferator-activated receptor γ; C/EBPα: CCAAT/enhancer-binding protein alpha; AMPK: AMP-activated protein kinase; SERBP: sterol regulatory element-binding proteins; LPL: lipoprotein lipase; FABP4: fatty-acid-binding proteins 4; FAS: fatty acid synthase; ATGL: adipose triglyceride lipase; TG: triglyceride; ACC1: acetyl-CoA carboxylase 1; ATPCL: ATP citrate lyase; CPT-1: carnitine palmitoyltransferase-1; FOXO1: forkhead box protein 1.

Fatty acids present in the cytoplasm have to send to the mitochondrial matrix for β-oxidation to produce ATP when fatty acids are taken up by cells. Because the inner membrane of mitochondria cannot penetrate long-chain fatty acids and acyl-CoA, fatty acids must be catalyzed by fatty acyl-CoA ligases to form a combination of fatty-acyl thioester and coenzyme A [72]. At this time, it is necessary to catalyze fatty acyl-carnitinem by CPT-1 on the outer membrane of mitochondrial membrane and pass through the outer membrane of mitochondrial membrane. CPT-2 on the inner membrane of mitochondria further converts fatty acyl-carnitinem into free carnitine and fatty acyl-CoA [72]. Fatty acyl-CoA is oxidized as soon as it enters the mitochondrial matrix. A series of steps begin with carbon atoms at the β position, each releasing two carbon fragments and existing as acetyl-CoA [73]. Each step includes four reactions, dehydrogenation, hydration, dehydrogenation, and thiolytic cleavage, in sequence [72]. Acyl-CoA produced either from β-oxidation or pyruvate oxidation enters the citric acid cycle and generates energy [72]. The mentioned pathway and role of each endocrine about the adipogenesis and adipolysis are shown in Figure 2 and Table 2.

### 4.2. Inflammation and Oxidative Stress Occurred with β-Oxidation

When β-oxidation occurs, reduced electron carriers are generated and ATP is generated via the oxidation phosphorylation of ADP [72]. Oxygen is the necessary substance when producing ATP in mitochondria; however, single electrons may escape and form a superoxide anion (O^2−^) with oxygen when electrons pass through the electron transfer chain. According to statistics, 1% of the oxygen used to generate ATP forms reactive oxygen species (ROS) [74]. Otherwise, it is generally considered that adipose tissue is a depot for lipids, but more and more studies demonstrate that adipose tissue also plays the role of an endocrine organ, producing signaling molecules called adipokines [75]. Adipokines mainly derived from white adipose tissue exhibit different functions in various physiological process, including regulating adipogenesis (e.g., chemerin), insulin sensitivity (e.g., adiponectin), feed intake (e.g., leptin and visfatin), energy homeostasis (e. g., adiponectin), and there are also adipokines with inflammatory functions (e.g., IL-6 and tumor necrosis factor-α (TNF-α)).

There are many factors that may cause chickens oxidative stress during the feeding process, such as environmental temperature, overcrowding and disease, which could promote excessive ROS and imbalance the antioxidant system of chickens [76,77]. However, obesity is also an oxidative stress factor since obesity keeps animals in a chronic inflammatory state. Obesity enhances superoxide formation and inhibits antioxidant-related enzymes [78]. Adipocytes produce ROS, and the level of ROS increases with the accumulation of visceral fat, which in turn stimulates the expression of inflammatory adipokines, increasing the number and activating macrophages in adipose tissue, thereby causing significantly obesity-induced adipose tissue inflammation [79]. Oxidative stress is also associated with many metabolic syndromes in animals, such as insulin resistance, hypertension, and negatively affects animal growth performance, leading to economic losses [80]. Ozata et al. [78] suggested obesity could be associated with oxidative stress and this might also relate to the occurrence of obesity-related diseases.

### 4.3. Adipose Metabolism-Related Hormone

Adipocytes also regulate endocrines, such as adiponectin, chemerin, visfatin, secreted by total adipocyte numbers and the situation of adipose accumulation. Adiponectin (also referred to as GBP-28, apM1, AdipoQ and Acrp30) is a 30-kDa adipocytokine hormone exclusively secreted from the adipose tissue, circulates as heavy, medium, and low molecular weight isoforms in mammals. The open reading frame of chicken adiponectin cDNA were 65–68% homologous to various mammalian adiponectin cDNAs [81,82]. It has many effects on metabolism, including the development of insulin resistance, antiatherogenic effects, and modulation of glucose and lipid metabolism. Hendricks et al. [83] demonstrated that adiponectin in chicken plasma and tissues was predominantly a heavy molecular weight multimer, and the level of plasma adiponectin was inversely related to abdominal fat pad mass. Yan et al. [84] used rosiglitazone (to activate adiponectin) and dexamethasone (to inhibit adiponectin) to treat broilers. Rosiglitazone increased serum adiponectin levels, and decreased with dexamethasone, while rosiglitazone decreased serum lipids, decreased lipid deposition in tissues (liver and muscle) and increased HDL-C, dexamethasone had the converse effect on serum lipids and fat distribution compared with rosiglitazone. In addition, chicken adipocyte differentiation trials showed a decrease in fat deposition and smaller lipid droplets in cells treated with adiponectin. The adiponectin was negatively correlated with fat deposition in chicken by decreasing the expression of fatty acid synthases (FAS) and C/EBPα and increasing the expression of ATGL.

Chemerin (also referred to as RARRES2 and TIG2) is an adipokine that modulates adipogenesis and immune function. Goralski et al. [85] pointed out adipocytes highly expressed chemerin and its receptor chemokine-like receptor 1 and both regulated the metabolism of lipid and glucose. Bozaoglu et al. [86] demonstrated that the expression of chemerin was positively correlated with obesity and increased during differentiation of 3T3-L1 adipocytes. Ress et al. [6] indicated that obese patients lose weight with a decrease in serum chemerin, and improve whole body low-grade inflammation and insulin sensitivity. However, chemerin and its receptor expressed in the adipose tissues, muscle and liver of poultry, and the plasma chemerin were negatively correlated with levels of cholesterol, triglycerides, phospholipids and fat percentage [6,87,88]. Chemerin has different correlations with lipid metabolism in mammals and poultry, but there is a lack of studies on poultry chemerin to make a clear discussion of the role of chemerin in the regulation of lipid metabolism.

Visfatin (also referred to as PBEF) is mainly produced in visceral adipose tissue, increases secreted plasma levels and expression, accompanied by obesity [89]. It also has several effects on appetite, muscle growth, glucose and lipid metabolism. Injection of visfatin in chicken could stimulate appetite and increase feed intake. Krzysik-Walker et al. [90] demonstrated that visfatin could regulate the expression of key myogenic transcription factors and thereby might influence muscle growth. Furthermore, the lateral hypothalamus and ventromedial hypothalamus were activated and deactivated by visfatin, respectively [91]. Piekarski et al. [92] found that supplementation of chenodeoxycholic acid in broiler chickens induced a reduction of feed intake and body weight, accompanied with a decrease in the expression of visfatin in the liver. In addition, there were effects on the increase of the expression of feeding-related hypothalamic neuropeptides, and a decrease in the mRNA levels of major hepatic lipogenic genes. According to the above studies, it suggesting that visfatin is a potent myogenesis, orexigenic, and lipid metabolism factor.

IL-6 is a cytokine which is secreted by adipocytes, and other sources including macrophage immune system cells, fibroblasts, endothelial cells, and skeletal muscle with a lot of biological activity in immune regulation, lipid metabolism, and inflammation [4,93]. Sindhu et al. [94] concluded that IL-6 expression in the adipose tissue was positive with obesity which might be a potential mechanism to induce metabolic inflammation. IL-6 attenuates insulin signaling, which is mediated by activation of JAK-STAT signaling in adipocytes and hepatocytes [95].

### 4.4. The AMP-Activated Protein Kinase (AMPK) Regulation of the Avian Appetite

In mammals, AMPK is one of the major factors of energy balance, and is suppressed by acetyl-CoA carboxylase (ACC), the mammalian target of rapamycin (mTOR), ATP level, glucose, leucine, and some kinds of lipid content in diet [96]. In their report, Song et al. [20] showed that the appetite regulated by AMPK was similar to that of a mammal. Fasting for 48 h increased the uric acid and nonesterified fatty acid level and decreased TG and glucose levels in serum compared to the control group [20,97]. Furthermore, fasting also increased the phosphorylation AMPK and increased the adipolysis and decreased the adipogenesis in chickens [20,97]. Previous research showed that AMPK controlled the energy balance in cells by regulating the uptake and metabolism of glucose and adipose [98]. As an upstream endocrine of AMPK, PPARα activated AMPK thereby stimulating FoxO1 to enter the nucleus to enhance the mRNA expression of ATGL. ATGL can hydrolyze triglycerides into fatty acids and diacylglycerol, thereby increasing β-oxidation [99]. Metformin activates AMPK in liver cells, also reduces the activity of ACC1 and induces the oxidation of fatty acids [100]. AMPK also inhibits de novo lipogenesis in the liver by reducing the performance of SREBP [101]. The expression of sterol regulatory element-binding proteins (SERBP) increases the activity of PPARγ and induces the differentiation of adipocytes. PPARγ is a key gene for adipogenesis and can activate C/EBPα, both of which can promote the expression of adipocyte differentiation-related factors, such as: FABP4, LPL and FAS [102].

However, PPAR does not seem to be a key gene for adipocyte differentiation in poultry, but rather FABP4 and C/EBP can induce adipocyte proliferation [101]. The FABP family can bind long-chain fatty acids or other hydrophobic molecules [103]. FABP4 has a role in affecting fatty acid uptake and transport [104]. LPL can induce fat accumulation, which can hydrolyze remnant-like lipoprotein particles (RLPs) and promote uptake of RLP, which may also lead to increased accumulation of lipids in cells [105]. Fatty acid synthase (FAS) is a key enzyme that synthesizes acetyl-CoA and malonyl-CoA into long-chain fatty acids [106].

ACC1 is highly expressed in the liver and adipose tissue, while ACC2 is mainly expressed in the heart and muscle. ACC2 is located in the mitochondria and ACC1 is located in the cytoplasm [107]. ATP citrate lyase catalyzes citric acid and CoA to form acetyl CoA, while ACC1 catalyzes the carboxylation of acetyl CoA to malonyl-CoA, and malonyl-CoA condenses with fatty acids under the catalysis of FAS [108]. Malonyl-CoA is also an inhibitor of mitochondrial carnitine palmitoyltransferase-1 (CPT-1) rate-limiting enzyme, while CPT-1 is responsible for transporting long-chain fatty acids into mitochondria [109]. In addition, in the research of Thupari et al. [110], it was found that the inhibition of FAS by cerulenin could also cause the increase of malonyl-CoA and thus inhibit the oxidation of CPT-1 and fatty acids in cells.

## 5. Effect of Rearing Pattern on Appetite and Metabolism Regulation

Animals can regulate appetite-related or metabolic-related hormone expression through dietary changes. In addition, microbiota could also be shaped by diet within 1 day [10]. In recent years, research on the intestinal microbiome has revealed that intestinal microorganisms have a considerable impact on animal obesity and health [10,11]. Therefore, the following sections discuss the effects on animal appetite and metabolism through the changing of feed composition.

### 5.1. Plant-Based Ingredients

It is well known that fiber content is positively related to animal intestinal health and increases the function of the gut barrier and decreases animal inflammatory levels [10]. However, to increase the growth of economical animals and benefit, the crude fiber content in feed is decreasing as possible in the past [111]. Recent research proves that increasing the crude fiber content appropriately does not downregulate the growth performance of poultry and may have some positive effects, including an increase in villus height and antioxidation ability and a decrease in inflammatory levels, [15,112,113].

Prebiotics are pure dietary fiber which cannot be digested by animals but can be digested by intestinal microbe flora and can promote the growth of probiotics in host intestines. Prebiotics can be a source for producing short-chain fatty acids by microbe flora and further decrease the colonization of pathogens [114]. Furthermore, as the main fermented product produced by microbiota and dietary fiber, butyrate downregulates the insulin receptor expression in the liver, abdominal adipose and subcutaneous adipose, but upregulates the insulin receptor expression and glucose intake in the muscles by the PI3K pathway [115]. Naked neck chicken fed with prebiotics derived from Saccharomyces cerevisiae cell walls including beta-D-glucan and mannan-oligosaccharides would also alter the composition of the cecum microbes [114]. High dietary fiber feed (5% lignocellulose, 19.4% crude protein and 6.8% crude fiber) could increase feed intake, 10% weight gain and decrease 38% visceral fat accumulation in both chickens (initial cross between inbred New Hampshire, high-growth) and White Leghorn (low-growth) lines compared to the low dietary fiber feed (0.8% lignocellulose, 19.4% crude protein and 4.1% crude fiber) [116]. Many researchers have shown that it is feasible to improve the intestinal microbial or body composition of the animal from the diet to promote its health or meet the preferences of consumers.

In appetite regulation, fiber can regulate the feed intake mainly by the fullness of the stomach (crop in poultry) [117]. Because of its water-holding capability, soluble fiber swells in the digestive tract, and increases the viscosity of chyme [117,118]. As the chyme increases with the passing of time, animals decrease their feed intake [117,118]. On the other hand, fermentable fiber also adjusts the feed intake by shifting animal microbiota and thereby regulating the metabolomics and animal endocrine [10,11,119]. Many agricultural processing byproducts have a high fiber composition, and most of these byproducts are treated by composting or cremation. However, more and more research shows that this agricultural waste could be one of the ingredients of animal feed and would decrease the cost of animal feeding [15,112,113]. In addition, to use directly in animal feed, fiber waste could also be fermented by probiotics as a postbiotic and increase the value of the fiber [120]. Overall, fiber has the potential to be used as a feed additive, however, the solubility and fermentability of fiber must be considered [117]. In addition to the benefits, there are limits to the use of fiber. With improper storage it may be easily contaminated with mycotoxins from agricultural byproducts. Excessive additions may indeed affect the growth of animals, so appropriate additions are still necessary [111].

Previous research showed that there are many antibacterial contents in plant extracts, such as essential oils (thymol and carvacrol, included). According to the report of Ultee et al. [121] 0.01 mM carvacrol can increase the membrane permeability of *Bacillus cereus*, and make hydrogen ions and potassium ions penetrate, eventually causing cells to lose membrane potential.

Carvacrol and thymol can also be antibacterial by altering the permeability of the cell membrane of *E. coli* [122]. However, the use of essential oils to reduce microorganisms can also have negative effects. Reis et al. [123] found that adding 1% of a phytogenic based on thymol, carvacrol and cinnamic aldehyde reduced the growth traits. The addition of essential oils may also kill the beneficial bacteria [123]. Even so, 600 mg/kg oregano essential oil (OEO) still can increase broiler average daily gain and decrease crypt depth and feed conversion rate [124]. This may be due to the fact that deeper crypts represent more intestinal cell proliferation and more energy consumption, which is not welcome in economic animals [124]. Under urgent and other stimuli, intestinal cells compensate for proliferation in response to stress [125]. The crypt depth is one of the indicators of whether the intestine is in a normal physiological state. The bactericidal effect can make the intestine a more stable environment, reduce the crypt depth and improve the nutritional utilization of broilers. Peng et al. [124] also mentioned that OEO can increase breast meat percentage and reduce abdominal fat rate. This may be because 80–85% of the body fat accumulated in adult birds comes from TG in plasma [16], while triglycerides in plasma are mainly at VLDL [17]. In previous research, dietary supplementation with OEO reduced the levels of triglycerides and VLDL in the serum [12,14]. When the TG decreases in animal serum, muscle cells and liver cells can avoid the risk of damage from fatty infiltration.

In addition to plant essential oils, different types of organic acids (butyric acid, fumaric acid, lactic acid) can also improve chicken body weight and feed conversion rate, and can increase villi height, serum calcium and phosphorus concentrations [67]. Addition of butyric acid can improve the nondigestible protein source of broilers, reduce the pH of gizzards and branched chain fatty acids (BCFA), and increase 6% ileal protein digestibility [126].

Epigallocatechin gallate (EGCG) is the most abundant catechin in green tea, which exhibits several effects of an antiobesity, antioxidant, and anti-inflammatory nature [13]. Huang et al. [127] supplemented EGCG to broilers which resulted in reducing the expression of SREBP-1c and its downstream FAS and SCD, indicating that EGCG has an effect on inhibiting fatty acid synthesis in the livers of broilers. Moreover, the decrease of stearoyl-CoA desaturase (SCD) expression contributes to reducing abdominal fat accumulation and serum triglycerides. Song et al. [77] indicated EGCG was effective in improving antioxidant capacity and anti-inflammatory ability on heat-stressed broilers through enhanced antioxidant and inflammatory related genes. In addition, EGCG also improved antioxidant capacity by increasing antioxidant enzyme activity. According to these, it is clear that plant extracts, essential oils and short chain fatty acids especially, can alter the microbe and serum nutrient type as well as alter the adipose metabolism patterns in poultry and further improve poultry health.

### 5.2. Probiotics

Probiotics can also promote chicken growth or reduce adipose accumulation. An addition of 0.8% *Saccharomyces Cerevisiae* increased egg production rates and lowered ileum pH in laying hens, which may due to a significant increase in lactic acid bacteria count [113,128]. *Saccharomyces Cerevisiae* addition could also decrease *Escherichia coli* (*E. coli*), *Klebsiella* sp., *Staphylococcus* sp., *Micrococcus* sp., *Campylobacter* sp., and *Clostridium perfringens* content [128]. A supplement of *B. subtilis* (10^5^ cfu/kg) could decrease broilers feed conversion rate and the emission of NH_3_ and H_2_S [68]. Furthermore, *Bacillus licheniformis* (5.6 × 10^9^ and 1.1 × 10^10^ cfu/mL) addition in water would both increase broilers’ body weight gains, improve feed conversion rates and further increase the protein and free amino acid content and decrease fat content in broilers’ breast meat [129].

### 5.3. Lighting Effect

Previous studies have shown that animals have certain growth curves and generated feedback after the weight fluctuations by external factors. Geary [1] used mathematical models to discuss the relationship between animal weight, appetite and animal growth feedback models. But mathematical models are beyond the scope of this report. Exogenous endocrines have been discussed in detail in the report above. Therefore, the following sections report the growth feedback caused by lighting adjustment.

Unlike mammals, poultry can receive light stimuli through their eyes and combs, which can affect their appetite or growth. Buyse et al. [130] pointed out that giving light rhythms (1L: 2D, 1L: 3D, 0.25L: 1.75D, 0.25L: 0.75D and 2L: 4D) could significantly reduce Cobb broilers’ feed intake (about 4.36%) and increase the broilers’ body weight (about 1.49%). Recent research has also shown that compared to 23L: 1D, 16L: 8D and 16L: 8D decreased feed intake and the number of the feed conversion rate. Furthermore, longer continuous dark time also increased the melatonin content in the broilers’ serum and the comfort behavior (preening, vertical wing-shaking, and wing-leg stretching included) [131].

Properly increasing the duration of continuous darkness or supplementing additional UVA can significantly reduce the ratio of tibial dyschondroplasia lesions, footpad dermatitis and hock burn score [7,131,132]. According to this, regulating the lighting time as well as type would affect avian feed intake and body weight. Furthermore, increased continuous dark time could also reduce animal injury and increase animal comfort behavior [7,131,132] to decrease the cytokine produce and increase the welfare of poultry, and thereby jump off the vicious circle of inflammatory-cortisol increased-fat accumulation-inflammatory.

## 6. Economic Animal Feeding Advice Based on Appetite Regulation and Fat Metabolism

High fiber feed ingredients are one of the inevitable byproducts of agricultural production. Increasing the utilization of fiber may not only reduce production costs, but also further enhance adipose metabolism and energy redistribution in poultry. In this way, poultry can have better health and lean meat output. In addition, many plant extracts, probiotics and moderate light and dark regulation also have the ability to improve poultry health and body composition. In order to maintain poultry welfare and production efficiency, according to the above report, we carefully recommend that increasing the fiber content in poultry diets would be beneficial.

## 7. Future Perspectives

We did our best to try to clarify known effects of poultry appetite regulation, energy metabolism homeostasis, and dietary patterns on poultry appetite and growth. However, there are still many causal relationships between appetite and fat metabolism that are unclear and need further research. Since the determination of poultry LEP in 2016, research about endocrine-regulated poultry appetite has been increasing. At present, the research on various endocrines on the regulation of poultry appetite has some foundation, but most of the research focuses on the regulation of poultry appetite by pure compounds, especially purified endocrines. However, in order to consider the economic cost of practical applications, the authors believe that the interaction between different feed additives on intestinal microbiota, metabolomics, physicochemical properties, and endocrine regulation on appetite of poultry should be discussed next, and according to the latest research, from the perspective of considering animal welfare, economic considerations, and effective use of the earth’s resources, more appropriate feeding recommendations should be proposed.

## 8. Conclusions

Adipose accumulation in poultry is accomplished through several metabolic pathways and may cause chicken health concerns and decrease consumer preference. Therefore, reducing adipose accumulation in poultry without reducing feed efficiency is a problem that must be faced. According to this report, the adjustment of environment and feed composition would both help to achieve this goal. Nevertheless, we also propose some functional feed additives and lighting methods that could physiologically regulate the adipose metabolism. In other words, to maximize the value of economic animal production and consider animal welfare at the same time, we recommend using agricultural byproducts with high fiber content. Higher-fiber diets are indeed expected to improve poultry production efficiency while reducing feed costs, so this feeding concept should be taken seriously and applied.

## Figures and Tables

**Table 1:** The function of appetite-related endocrines in poultry.

Endocrine.	Animal	Age	Effect	Methods ^1^	References
Leptin	Sanhuang broiler breeder eggs	In ovo	Increases feed intake in 21D Decrease GR expression	In ovo, 0.5 or 5 μg	[32]
Leptin	Cobb broiler	4-day-old	Does not affect feed intake	i.c.v., 0.3–3 nmol	[37]
Leptin	Ross broiler	9-day-old	Does not affect feed intake	i.p., 0.5 mg/kg	[35]
Leptin	ISA layer	9-day-old	Decreases feed intake	i.p., 0.5 mg/kg	[35]
Leptin	broiler	4-week-old	Decreases feed intake	i.c.v., 2.5–10 μg	[34]
Leptin	Leghorn	7-week-old	Decreases feed intake	i.c.v., 2.5–10 μg	[34]
NPY	Hubbard X Cobb 500 broiler	4-week-old	Increases high carbohydrate and protein intake	i.c.v., 0.2–2 nmol	[8]
NPY	Adipose cell	14-day-old	Decreases adipolysis-related-mRNA expression	In vitro, 1–100 nM	[67]
NPY	Chunky broiler and Leghorn	1 to 8-day-old	Increases feed intake	i.c.v., 0.2–0.4 μg	[26]
α-MSH ^2^	Leghorn and chunky broiler	8-day-old	Decrease feed intake	40–400 pmol	[20]
β-MSH ^2^			Decreases Leghorn feed intake		
γ-MSH ^2^			Does not affect the feed intake		
α-MSH	Cobb-500 broilers	4-day-old	Decreases NPYR1 ^3^ mRNA expression	i.c.v., 0.12 nmol	[24]
Oxytocin	Cobb-500 broilers	4-day-old	Decreases feed intake and increases adipolysis	i.c.v., 0–10 nmol	[43]
GnIH ^4^	Julia male layer chicks	14-day-old	Increases feed intake and	i.c.v., 0–7.8 nmol	[39]
CORT ^5^	Hy-line brown layer	24-week-old	Increases serum glucose and insulin level and decreases TG ^6^ and NEFA ^7^ content	s.c., 2 mg/kg	[68]
Insulin	White Leghorn	8-day-old	Increases POMC ^8^, CART ^9^, α-MSH and CRF ^10^ mRNA expression	i.c.v., 0.1–10 μg	[50]

^1^ i.c.v.: Intracerebroventricular injection; i.p.: intraperitoneal injection; s.c.: subcutaneous injection. ^2^ MSH: melanocyte stimulating hormone. ^3^ NPYR1: neuropeptide Y receptor 1. ^4^ GnIH: gonadotropin inhibitory hormone. ^5^ CORT: corticosterone. ^6^ TG: triglyceride. ^7^ NEFA: monesterified fatty acids. ^8^ POMC: pro-opiomelanocortin. ^9^ CART: cocaine and amphetamine regulated transcript. ^10^ CRF: corticotropin-releasing factor.

**Table 2:** The role of key endocrines that are involved in adipose metabolism ^1^.

Endocrines ^2^	β-Oxidation	Endocrines ^3^	β-Oxidation
PPARα	+	SERBP	+−
AMPK	+	PPARγ	+−
CPT-1	+	C/EBPα	+−
ACC1	−	FAS	+−
Malonyl-CoA	−	ATGL	+−

^1^ “+”: accelerates β-oxidation; “−”: suppresses β-oxidation; “+−”: indirectly promotes β-oxidation (by increasing fatty acids amount). ^2^ PPARα: peroxisome proliferator-activated receptor α; AMPK: AMP-activated protein kinase; CPT-1: carnitine palmitoyltransferase-1; ACC1: acetyl-CoA carboxylase. ^3^ SERBP: sterol regulatory element-binding proteins; PPARγ: peroxisome proliferator-activated receptor γ; C/EBP-α: CCAAT/enhancer-binding protein alpha; FAS: fatty acid synthase; ATGL: adipose triglyceride lipase.

## Abbreviations/Nomenclature

ACC	acetyl-CoA carboxylase
ACTH	adrenocorticotropic hormone
AgRP	agouti-related protein
AMPK	AMP-activated protein kinase
ATGL	adipose triglyceride lipase
BCFA	branched chain fatty acids
C/EBP-α	CCAAT/enhancer-binding protein alpha
CART	cocaine and amphetamine regulated transcript
cGH	chicken growth hormone
CORT	corticosterone
CPT	carnitine palmitoyltransferase
CRF	corticotropin-releasing factor
CRH	corticotropin-releasing hormone
EGCG	epigallocatechin gallate
FABP	fatty-acid-binding proteins
FAS	fatty acid synthase
FoxO1	forkhead box protein
FSH	follicle stimulating hormone
GH	growth hormone
GLUT	glucose transporter
GnIH	gonadotropin inhibitory hormone
GnRH	gonadotropin-releasing hormone
GR	glucocorticoid receptor
HDL	high density lipoprotein
GSH-Px	glutathione peroxidase
HMW	heavy molecular weight
HPA	hypothalamic--pituitary--adrenal
IL-6	Interleukin
JAK2-dependent	Janus kinase 2-dependent
JAK-STAT	Janus kinase/signal transducers and activators of transcription
LDL	low density lipoprotein
LEP	leptin
LEPR	leptin receptor
LH	luteinizing hormone
LPL	lipoprotein lipase
MAPK	mitogen-activated protein kinase
MRAP	melanocortin receptor accessory proteins
MSH	melanocyte stimulating hormone
NEFA	monesterified fatty acids
NPR	neuropeptide Y receptor
NPY	neuropeptide Y
OEO	oregano essential oil
OXT	oxytocin
OXTR	oxytocin receptor
PI3K	Phosphoinositide 3-kinase
POMC	pro-opiomelanocortin
PPAR	peroxisome proliferator-activated receptor
PVN	paraventricular nucleus
RARRES	retinoic acid receptor responder
RLPs	remnant-like lipoprotein particles
ROS	reactive oxygen species
SCD	stearoyl-CoA desaturase
SERBP	sterol regulatory element-binding proteins
STAT3	signal transducer and activator of transcription 3
TG	triglyceride
TNF-α	tumor necrosis factor-α
VLDL	very low density lipoprotein.
